# Supplemental thiamine for the treatment of acute heart failure syndrome: a randomized controlled trial

**DOI:** 10.1186/s12906-019-2506-8

**Published:** 2019-05-06

**Authors:** Howard A. Smithline, Michael Donnino, Fidela S. J. Blank, Richard Barus, Ryan A. Coute, Alexander B. Knee, Paul Visintainer

**Affiliations:** 1 Department of Emergency Medicine, Baystate Medical Center, University of Massachusetts Medical School - Baystate, 759 Chestnut Street, Springfield, MA 01199 USA; 2 Department of Emergency Medicine, Beth Israel Deaconess Medical Center, Harvard Medical School, One Deaconess Road, Rosenberg 2, Boston, MA 02215 USA; 3 Department of Emergency Medicine, Baystate Medical Center, 759 Chestnut Street, Springfield, MA 01199 USA; 4 Department of Nursing, Baystate Medical Center, 759 Chestnut Street, Springfield, MA 01199 USA; 5 University of Alabama Hospital, 1802 6th Ave S, Birmingham, AL 35233 USA; 6 Baystate Medical Center, Office of Research, Epidemiology/Biostatistics Research Core, University of Massachusetts Medical School, Baystate, 759 Chestnut Street, Springfield, MA 01199 USA

**Keywords:** Acute heart failure, Thiamine, Dyspnea

## Abstract

**Background:**

The purpose of this pilot study was to determine if a definitive clinical trial of thiamine supplementation was warranted in patients with acute heart failure. We hypothesized that thiamine, when added to standard of care, would improve dyspnea (primary outcome) in hospitalized patients with acute heart failure. Peak expiratory flow rate, type B natriuretic peptide, free fatty acids, glucose, hospital length of stay, as well as 30-day rehospitalization and mortality were pre-planned secondary outcome measures.

**Methods:**

This was a blinded experimental study at two urban academic hospitals. Consecutive patients admitted from the Emergency Department with a primary diagnosis of acute heart failure were recruited over 2 years. Patients on a daily dietary supplement were excluded. Randomization was stratified by type B natriuretic peptide and diabetes medication categories. Subjects received study drug (100 mg thiamine or placebo) in the evening of their first and second day. Outcome measures were obtained 8 h after study drug infusion. Dyspnea was measured on a 100-mm visual analog scale sitting up on oxygen, sitting up off oxygen, and lying supine off oxygen with 0 indicating no dyspnea. Data were analyzed using mixed-models as well as linear, negative binomial and logistic regression models to assess the impact of group on outcome measures.

**Results:**

Of 130 subjects randomized, 118 had evaluable data (55 in the control and 63 in the treatment groups), 89% in both groups were adjudicated to have primarily AHF. Thiamine values increased significantly in the treatment group and were unchanged in the control group. One patient had thiamine deficiency. Only dyspnea measured sitting upright on oxygen differed significantly by group over time. No change was found for the other measures of dyspnea and all of the secondary measures.

**Conclusions:**

In mild-moderate acute heart failure patients without thiamine deficiency, a standard dosing regimen of thiamine did not improve dyspnea, biomarkers, or other clinical parameters.

**Trial registration:**

ClinicalTrials.gov: NCT00680706, May 20, 2008 (retrospectively registered).

**Electronic supplementary material:**

The online version of this article (10.1186/s12906-019-2506-8) contains supplementary material, which is available to authorized users.

## Background

Acute heart failure syndrome is a common and costly medical condition. It accounts for more than one million hospitalizations annually in the United States. Eighty percent of these patients presenting to the emergency department are admitted. Their median hospital length of stay is 4 days, the in-hospital mortality rate is 4%, and the 30-day mortality rate is 12% [1].

The heart derives its energy primarily from glucose and fatty acid ß-oxidation [2]. During the progression of the disease there is a decrease in adenosine triphosphate (ATP) production in both systolic and diastolic heart failure [3, 4]. Shifting substrate utilization from fatty acid to glucose oxidation increases the amount of ATP produced per molecule of oxygen utilized [5]. Shifting substrate utilization from fatty acid to glucose oxidation can be accomplished by either stimulating glucose metabolism or inhibiting fatty acid metabolism. This approach has been shown to be effective for treating cardiac ischemia in animal studies as well as small human studies [6]. However, this approach has both theoretical and real limitations secondary to side effects of these agents, known toxicity associated with inherited abnormalities in fatty acid metabolism, and limited benefits when added to conventional therapy [6].

A better approach may be to correct abnormalities that cause a deleterious shift in myocardial substrate utilization to fatty acid metabolism by blocking glucose metabolism. Thiamine insufficiency is a possible example of this type of abnormality.

Patients with heart failure are at risk for thiamine deficiency [7]. Thiamine is a necessary cofactor for pyruvate dehydrogenase. Inhibition of this enzyme is the presumed primary mechanism by which thiamine deficiency causes wet beriberi, high output heart failure [8, 9]. However, it is unknown if low thiamine levels contribute to the severity of heart failure in patients without overt beriberi. If low thiamine levels contribute to heart failure severity then supplementation should be beneficial to patients admitted to the hospital with acute heart failure. The purpose of this pilot study was to determine if a definitive clinical trial of thiamine supplementation was warranted in patients with acute heart failure. This study was novel in that it used a patient centered outcome, dyspnea, as the primary outcome measure. It was also novel in that it studied patients with acutely decompensated disease.

## Methods

### Study design

This was a stratified block randomized double-blind placebo controlled study approved by the Institutional Review Board at both hospitals. Subjects were stratified on diabetes medication status (none, oral only, insulin) and initial NT-proBNP value (≤ 1500, 1501–4000, 4001–10,000, > 10,000 pg/ml). Separate randomization tables were developed for each of the 12 strata (four NT-proBNP and three diabetes medication strata). Subjects were stratified on diabetes status because of its impact on glucose and fatty acid metabolism. The NT-proBNP quartile values from the PRIDE study were used to define these strata [10].

### Study setting and population

Subjects identified in the Emergency Department being admitted for acute heart failure at two academic tertiary care medical centers on weekdays between 8 AM and 10 PM were screened for enrollment. Inclusion criteria included history of heart failure on a loop diuretic, worsening dyspnea over the past 24 h, dyspnea at time of enrollment, radiographic cephalization of vessels, elevated NT-proBNP (> 450 pg/ml), age > 18 years, a primary admitting diagnosis of acute heart failure. Exclusion criteria included renal failure on dialysis, severe valvular disease, acute myocardial infarction diagnosed by either ECG or initial troponin, ventricular arrhythmia (ventricular tachycardia or fibrillation), supraventricular arrhythmia (atrial fibrillation / flutter) with a ventricular rate > 120 beats per minute, taking thiamine or fatty acid supplementation within the past 2 weeks, pregnant.

### Study protocol

**Fig. 1:**
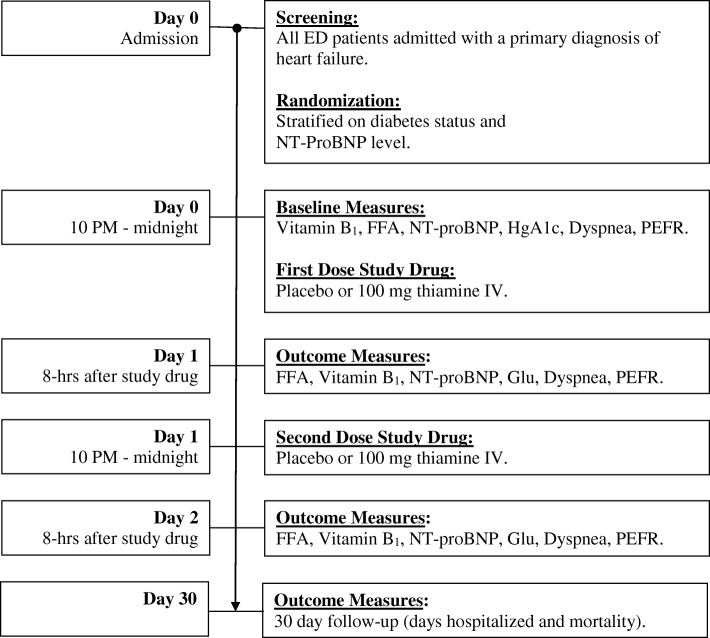
Study Design

Subjects that met the above criteria and gave written consent were randomized to either the Treatment arm (thiamine) or the Control arm (placebo). Randomization was performed by the central research pharmacy. The first dose of the study drug was given between 10 PM and midnight on the day the patient was admitted to the hospital and the second dose was given at the same time the subsequent evening. Baseline dyspnea measures and blood tests were obtained immediately prior to study drug infusion. Outcome measures were obtained 8 h after the study drug was given and after an overnight fast. One month later, 30-day outcome measures were obtained by review of the subject’s medical record, telephone call, and review of the social security death registry (Fig. 1).

The study drug consisted of 100 mg of thiamine hydrochloride in a solution of 50 ml of 5% dextrose in water. Placebo consisted of the same quantity of the dextrose solution. The study drug was infused over 20 min. Patients were otherwise treated at the discretion of the clinical team following the hospital’s clinical practice guideline for acute heart failure.

### Measurements

Dyspnea severity was the primary outcome measure. Dyspnea was chosen because it is a patient centered outcome measure and it is the primary symptom of acute decompensated heart failure that brings patients to the emergency department [11]. As such, improvement in dyspnea has been recommended as an important endpoint for clinical trials [11]. The subjects assessed their dyspnea severity using a 10-cm visual analog scale (VAS) in up to three positions as tolerated at each time point. Position-1: sitting upright on supplemental oxygen. Position-2: sitting upright off oxygen. Position-3: lying supine off oxygen. Subjects not on supplemental oxygen skipped Position-1. Subjects acclimated at each position for 5 min. Each VAS was measured to the nearest millimeter with a score of 0 indicating no dyspnea and a score of 100 indicating very severe dyspnea. This multi-position approach has been described by Pang et al. as a Provocative Dyspnoea Assessment (PDA); however, in that paper, dyspnea was measured using a 5-point numeric scale [11]. The traditional method for calculating the dyspnea severity score uses the raw VAS score from first position of the PDA. This was the primary method we chose for this study. However, the amount of oxygen supplementation was not controlled in this study and it could range from high flow oxygen with a non-rebreather mask to room air. To control for this variation we also calculated the dyspnea severity score by only using the raw VAS score from position-2 (upright while on no supplemental oxygen). Finally, we also calculated the dyspnea severity score by combining scaled VAS scores (VAS-PDA) from the three positions. The scaling was performed by adding 200 points to position 1 scores; 100 to position 2 scores and 0 to position 3 scores. This created a summed scaled score that ranged from best dyspnea (0 at all 3 positions = 0) to worst (100 at all 3 positions = 300). For this method, the VAS scores from the three positions were scaled by adding 0 to the VAS scores from Position-1, adding 100 to the VAS scores from Position-2, and adding 200 to the VAS scores from Position-3. The scaled score at the last completed position was used.

In addition to dyspnea severity, N-terminal prohormone of brain natriuretic peptide (NT-proBNP) was measured to assess heart failure severity. Free fatty acids (FFA) and glucose was measured to assess change in metabolism. Peak expiratory flow rate (PEFR) was measured as an alternate method for assessing respiratory status.

Additionally, whole blood thiamine levels were measured. For all analyses the whole blood thiamine level was divided by the hemoglobin concentration. Hemoglobin A1c was also measured at baseline to assess adequacy of diabetes control. Thiamine was measured using liquid chromatography/tandem mass spectrometry and FFA was measured using spectrophotometry. Both tests were performed by LabCorp, Inc. The other assays were performed by the clinical laboratory at Tufts Medical Center. At 30 days the medical record was assessed and subjects were contacted to determine if they were re-hospitalized, duration of hospitalization and mortality.

For purposes of a post-hoc analysis, the medical records of all subjects were reviewed after hospital discharge by two senior emergency medicine attendings to determine if the subjects dyspnea was primarily caused by acute heart failure. In cases of disagreement, a committee consisting of the two reviewers met to reach consensus.

### Data analysis

A total sample size of 150 subjects (25 subjects in each diabetes strata for each treatment arm) was originally planned as the maximum number of patients that could be enrolled within the financial and time constraints of the grant.

We initially evaluated descriptive measures (numbers and percents for categorical; means, standard deviations and ranges for continuous) for our study variables to assess distributions, outliers and screen for data entry errors. To determine if subjects were balanced across study treatment group, we evaluated subject characteristics, baseline labs and baseline outcomes by thiamine supplementation. Differences were evaluated primarily based on clinical importance however we also used statistical tests (Fisher exact test for categorical data and unpaired t-tests for continuous data) to screen for potential associations. In addition, the above variables were evaluated for associations with our primary and secondary study outcomes using linear (change over time), negative binomial (length of stay in days) and logistic (30-day readmission and 30-day mortality) regression. Models of 30-day readmission excluded deceased subjects. No modeling was performed regarding 30-day mortality due to the limited number of deaths.

We utilized linear mixed models to evaluate change in our physiologic outcomes over time. The advantage of using this method is the ability of the model to utilize all available data as opposed to casewise deletion of subjects missing outcomes at certain time points. Unadjusted models included design effects (diabetes category and NT-proBNP quartile) as well as site. Change over time was evaluated using an interaction term between treatment group and time period. Hypothesis testing was conducted using either likelihood ratio tests or Wald tests (if robust standard errors were used) of the full and reduced models in regards to the interaction term. Results were reported as marginal means and 95% confidence intervals. Sidak adjustments were used when multiple comparisons were conducted.

To develop our multivariate models and assess for potential confounding, we evaluated differences between covariates and treatment groups as well as covariates and our outcome measures (at baseline if a repeated measure) using the methods described above. Estimates were evaluated based on clinically meaningful differences, as well as with statistical tests. We used a liberal *p*-value of less than 0.25 to help screen for potential confounders. Covariates were selected based on current literature, physiology and whether there was a potential association between the covariate and both exposure and baseline outcome. In addition to design effects (which remained in the model regardless of significance testing) all multivariable models initially included baseline measures for the outcome (for repeated measures), thiamine, body mass index, peak expiratory flow rate, systolic blood pressure, left ventricular ejection fraction (LVEF) greater or equal to 50%, and history of myocardial infarction. With the goal of achieving a parsimonious model, we iteratively removed the remaining terms initially above the 0.25 level followed by the 0.10 level. As this study was designed as a pilot, and the sample was small, covariates were retained at the 0.10 level as these terms were suggestive of potentially useful adjustments. For our physiologic measures, regardless of significance testing, we also retained baseline outcomes and thiamine in all models. Full and reduced models were evaluated using likelihood ratio tests or Wald tests. Model fit and diagnostics were evaluated before finalizing the models and creating graphical representations of the associations. All models were evaluated on our intention to treat group as well as those with adjudicated acute heart failure syndrome. Data analysis was conducted using Stata v 12.1 [12].

## Results

**Fig. 2:**
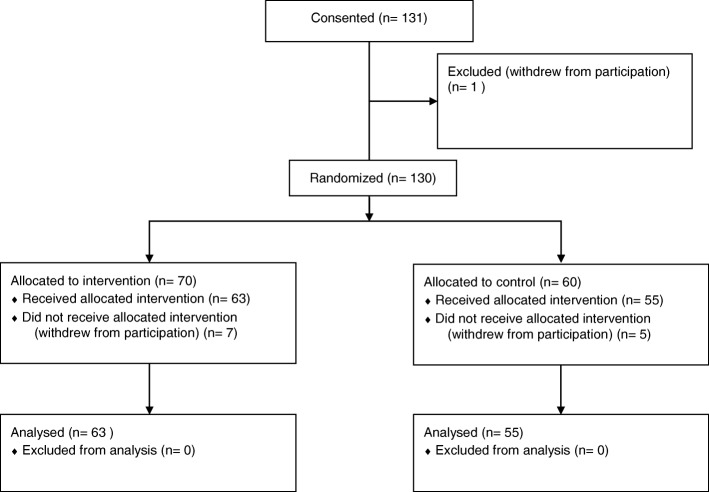
Flow Chart

A total of 131 subjects consented over 2 years. One subject was dropped after consent but before randomization. Of the 130 subjects randomized, 60 were allocated to the control group and 70 to the thiamine group. Twelve subjects were not included in the analysis because they were dropped from the study before the first outcome measures were obtained. This left 55 subjects in the control group and 63 subjects in the thiamine group with evaluable data. Of the 55 subjects in the control group, 49 (89%) were adjudicated to have primarily acute heart failure. Of the 63 subjects in the thiamine group, 56 (89%) were adjudicated to have primarily acute heart failure (Fig. 2). Subject characteristics are shown in Table 1.

**Table 1:** Subject Characteristics

	Control (*n* = 55)	Treatment (*n* = 63)
Female	43.64%	49.18%
Age [mean (sd)] (years)	73.25 (13.29)	72.64 (13.23)
Body Mass Index [mean (sd)] (kg/m^2^)	32.98 (9.05)	30.31 (8.41)
Race		
Caucasian	81.82%	75.41%
Black	18.18%	19.67%
American Indian or Alaska Native	0%	3.28%
Other	0%	1.64%
Hispanic Ethnicity	0.00%	8.20%
Diabetes	60.38%	58.33%
Diabetes Medications		
None	49.06%	45.76%
Oral Only	26.42%	18.64%
Insulin Only	18.87%	28.81%
Insulin and Oral	5.66%	6.78%
Past Medical History		
MI	26.92%	50.88%
CAD	38.46%	52.63%
Angina	21.15%	27.27%
Left Ventricular Ejection Fraction (LVEF) [mean (sd)]	43.01% (18.99%)	40.84% (19.16%)
LVEF ≥50%	48.15%	42.86%
New York Heart Association (NYHA) Functional Classification		
1	0.00%	1.69%
2	17.31%	13.56%
3	73.08%	67.80%
4	9.62%	16.95%
Goldman Specific Activity Scale Functional Classification		
1	5.77%	3.51%
2	11.54%	17.54%
3	63.46%	61.40%
4	19.23%	17.54%
Initial Laboratory Values [mean (sd)]		
NT-proBNP (ng/mL)	5555 (6845)	8656 (12,780)
NT-proBNP Quartiles		
≤ 1500	22.22%	17.46%
1501–4000	38.89%	42.86%
4001–10,000	22.22%	12.70%
≥ 10,001	16.67%	26.98%
Hgb (g/dL)	11.97 (1.75)	11.79 (1.65)
Hct (%)	37.34 (5.13)	36.67 (4.80)
Na (mEq/L)	138.78 (5.17)	138.70 (3.64)
K (mEq/L)	4.42 (1.07)	4.37 (0.89)
Glu (mg/dL)	163.81 (78.39)	155.43 (88.56)
Cl (mEq/L)	100.98 (6.27)	101.90 (4.55)
CO_2_ (mEq/L)	26.54 (5.22)	25.98 (5.01)
BUN (mg/dL)	30.29 (17.16)	30.83 (16.54)
Cr (mg/dL)	1.37 (0.58)	1.32 (0.56)
Troponin 1 (mg/mL)	0.03 (0.05)	0.03 (0.04)
Troponin 2 (mg/mL)	0.04 (0.07)	0.04 (0.06)
HbA1c (%)	6.93 (1.27)	6.94 (1.70)
Mg (mEq/L)	1.69 (0.29)	1.67 (0.28)
Thiamine (whole blood) (nmol/L)	125.06 (33.86)	125.44 (36.18)
Thiamine (plasma) (nmol/L)	27.40 (17.35)	30.80 (34.10)
Free fatty acid (mEg/L)	0.46 (0.29)	0.46 (0.33)
Vital Signs [mean (sd)]		
Systolic blood pressure (mmHg)	140.64 (29.77)	131.68 (34.13)
Diastolic blood pressure (mmHg)	71.49 (17.38)	66.15 (17.53)
Heart rate (beats/min)	82.50 (17.06)	79.94 (16.94)
Initial Peak Expiratory Flow Rate (PEFR) (L/min)	200.13 (96.65)	173.76 (77.68)

### Thiamine

Whole blood thiamine levels (units: nmol/L/g Hgb) were computed from a mixed model at baseline, time-1 and time-2. Values remained constant for the control group but increased significantly for the thiamine group (Table 2). Only 1 patient (thiamine group) was deficient in thiamine as defined by a whole blood thiamine level < 66.5 nmol/L.

**Table 2:** Whole Blood Thiamine divided by Hemoglobin (nmol/L/g Hgb) by Time (*n* = 117)

	Control	Treatment	Difference	*p-value (Sidak adjusted)*
Baseline	10.70 (4.82–16.57)	10.75 (5.26–16.24)	0.05 (−9.74–9.85)	0.990
Time 1	10.36 (4.53–16.19)	51.67 (46.14–57.20)	41.31 (31.52–51.10)	< 0.001
Time 2	10.53 (4.19–16.88)	60.21 (54.09–66.34)	49.68 (38.94–60.42)	< 0.001

Values are means with 95% CI from linear mixed model

### Physiologic outcome measures

All measures are initially described across all time periods before and after adjustment (Table 3). All models were also run with data restricted to subjects adjudicated as having primarily acute heart failure, however all of these results were essentially unchanged from models utilizing all subjects (data not shown).

**Table 3:** Physiologic outcomes by thiamine supplementation over time

Measure	Baseline		Day 1		Day 2		*p-value (model)*
	Control	Treatment	Control	Treatment	Control	Treatment	
VAS-1st Position (mm)							
Unadjusted (*n* = 116)^*^	31 (24–38)	26 (19–33)	17 (12–22)	22 (16–28)	17 (10–24)	21 (15–27)	*0.015*
Adjusted (*n* = 104)^**a, b^	31 (28–33)	28 (25–30)	16 (11–21)	24 (20–29)	15 (8–22)	26 (19–32)	*0.020*
VAS-2nd Position (mm)							
Unadjusted (*n* = 103)^*^	32 (24–40)	25 (18–32)	19 (13–25)	19 (14–25)	16 (9–23)	18 (13–23)	*0.132*
Adjusted (*n* = 83)^** b, c^	30 (27–34)	27 (24–29)	17 (12–22)	21 (17–24)	11 (5–18)	20 (15–25)	*0.093*
VAS-PDA (mm)							
Unadjusted (*n* = 116)^*^	103 (78–128)	80 (59–101)	78 (54–102)	59 (40–78)	60 (34–85)	64 (42–85)	*0.159*
Adjusted (*n* = 97)^** a, c^	85 (76–95)	78 (69–87)	64 (42–86)	61 (47–74)	45 (20–70)	63 (44–82)	*0.226*
PEFR (L/min)							
Unadjusted (*n* = 115)^*^	203 (180–226)	173 (152–194)	206 (183–229)	163 (141–184)	206 (183–230)	167 (146–189)	*0.237*
Adjusted (*n* = 103)^** b, d^	188 (178–197)	183 (174–192)	193 (184–202)	172 (163–181)	195 (184–205)	178 (168–188)	*0.124*
NT-proBNP (ng/ml)							
Unadjusted (*n* = 113)^*^	0.42 (0.32–0.52)	0.52 (0.35–0.68)	0.45 (0.30–0.61)	0.41 (0.28–0.54)	0.27 (0.21–0.33)	0.37 (0.26–0.48)	*0.223*
Adjusted (*n* = 100)^** c^	0.46 (0.42–0.50)	0.48 (0.42–0.54)	0.42 (0.34–0.51)	0.34 (0.27–0.41)	0.31 (0.24–0.38)	0.32 (0.24–0.41)	*0.209*
FFA (mEq/ml)							
Unadjusted (*n* = 114)^*^	0.46 (0.38–0.54)	0.47 (0.39–0.56)	0.68 (0.62–0.74)	0.65 (0.59–0.71)	0.62 (0.55–0.68)	0.71 (0.64–0.78)	*0.086*
Adjusted (*n* = 102)^** a, b^	0.46 (0.41–0.50)	0.45 (0.41–0.50)	0.69 (0.62–0.75)	0.63 (0.56–0.69)	0.62 (0.54–0.69)	0.68 (0.61–0.75)	*0.087*
Glucose (mg/dl)							
Unadjusted (*n* = 117)^*^	166 (146–186)	155 (135–175)	121 (110–132)	112 (102–121)	118 (110–126)	117 (107–126)	*0.491*
Adjusted (*n* = 113)^**^	161 (150–173)	157 (145–169)	120 (109–130)	113 (99–126)	117 (109–126)	119 (108–131)	*0.515*

Values are means with 95%CI. *P*-values are from a test of the treatment*time interaction term in the mixed model

^*^Includes design variables: site, diabetes medication (self-report) and NT-proBNP quartile

^**^Additional adjustments for: baseline values of the outcome, thiamine, BMI^a^, LVEF> = 50% ^b^, PEFR ^c^, systolic BP ^d^

Dyspnea severity, as measured using the primary method (position #1) in the unadjusted model, improved to a greater degree in the control group compared to the thiamine group. The VAS score (1st Position- unadjusted) decreased (improved) by 14 mm (95%CI: 5 mm to 23 mm) from baseline to day 2 in the control group compared to the thiamine group that decreased by 5 mm (95%CI: -5 mm to 14 mm). In the unadjusted model, the difference between the control and treatment groups over time was statistically significant, *p* = 0.015. This change remained statistically significant in the adjusted model, *p* = 0.020. However, there was no statistical difference between the two groups on change in VAS score (2nd Position) or change in VAS score (PDA) baseline and day 2 in both the unadjusted (*p* = 0.132 and *p* = 0.159 respectively) and adjusted models (*p* = 0.093 and *p* = 0.226 respectively). The VAS score (2nd Position-unadjusted) decreased (improved) by 16 mm (95%CI: 6 mm to 26 mm) from baseline to day 2 in the control group and decreased by 7 mm (95%CI: -2 mm to 16 mm) in the thiamine group. The VAS score (PDA-unadjusted) decreased (improved) by 44 mm (95%CI: 9 mm to 79 mm) from baseline to day 2 in the control group and decreased by 16 mm (95%CI: -6 mm to 39 mm) in the thiamine group. The mean differences and 95% confidence intervals from baseline to Time 1 and 2 for both the unadjusted and adjusted models are shown in Table 4.

**Table 4:** Mean difference and 95% CI over time compared to baseline

Measure	Delta Baseline to Time 1		Delta Baseline to Time 2	
	Control	Treatment	Control	Treatment
VAS-1st Position (mm)				
Unadjusted^*^	−14 (− 21 to −7)	−4 (− 10 to 2)	−14 (− 23 to −5)	− 5 (− 14 to 5)
Adjusted^**a, b^	−15 (− 22 to − 7)	−4 (− 10 to 3)	−16 (− 26 to −6)	−2 (− 12 to 8)
VAS-2nd Position (mm)				
Unadjusted^*^	−13 (− 21 t0–5)	−5 (− 11 to 1)	−16 (− 26 to − 6)	−7 (− 16 to 2)
Adjusted^** b, c^	−13 (− 22 to − 5)	−6 (− 12 to 0)	−19 (− 30 to − 8)	− 7 (− 16 to 3)
VAS-PDA (mm)				
Unadjusted^*^	−25 (− 56 to 5)	−22 (− 41 to − 2)	− 44 (− 79 to − 9)	−16 (− 39 to 6)
Adjusted^** a, c^	− 21 (− 55 to 12)	−18 (− 36 to 1)	−40 (− 79 to − 1)	− 15 (− 40 to 10)
PEFR (L/min)				
Unadjusted^*^	4 (−12 to 19)	− 10 (− 25 to 4)	4 (− 13 to 20)	− 6 (− 21 to 10)
Adjusted^** b, d^	5 (− 10 to 21)	− 11 (− 26 to 3)	7 (− 9 to 23)	−5 (− 20 to 10)
NT-proBNP (ng/ml)				
Unadjusted^*^	0.03 (−0.16 to 0.23)	−0.11 (− 0.23 to 0.02)	−0.15 (− 0.28 to − 0.02)	−0.15 (− 0.30 to − 0.01)
Adjusted^** c^	−0.03 (− 0.14 to 0.07)	−0.14 (− 0.27 to − 0.01)	−0.14 (− 0.27 to − 0.02)	−0.16 (− 0.31 to − 0.002)
FFA (mEq/ml)				
Unadjusted^*^	0.22 (0.11 to 0.32)	0.18 (0.07 to 0.30)	0.16 (0.05 to 0.27)	0.24 (0.13 to 0.35)
Adjusted^** a, b^	0.23 (0.12 to 0.35)	0.17 (0.05 to 0.30)	0.16 (0.03 to 0.28)	0.23 (0.11 to 0.35)
Glucose (mg/dl)				
Unadjusted^*^	−45 (−70 to − 21)	−43 (− 73 to − 14)	−48 (− 72 to − 24)	−38 (− 64 to − 13)
Adjusted^**^	−42 (− 65 to − 18)	−44 (− 74 to − 14)	−44 (− 67 to − 21)	−37 (− 63 to − 11)

Values are mean difference and Sidak adjusted 95% Confidence Intervals

^*^Includes design variables: site, diabetes medication (self-report) and NT-proBNP quartile

^**^Additional adjustments for: baseline values of the outcome, thiamine, BMI^a^, LVEF> = 50% ^b^, PEFR ^c^, systolic BP ^d^

There was no statistically significant difference between the two groups on change in PEFR from baseline to day 2 in both the unadjusted (*p* = 0.237) and adjusted (*p* = 0.124) models. The PEFR (unadjusted) value increased (improved) by 4 L/min (95%CI: -13 L/min to 20 L/min) from baseline to day 2 in the control group and decreased (worsened) by 6 L/min (95%CI: -10 L/min to 21 L/min) in the thiamine group. The mean differences and 95% confidence intervals from baseline to Time 1 and 2 for both the unadjusted and adjusted models are shown in Table 4.

There was no statistically significant difference between the two groups on change in NT-proBNP from baseline to day 2 in both the unadjusted (*p* = 0.223) and adjusted (*p* = 0.209) models. The NT-proBNP (unadjusted) value decreased (improved) by 0.15 ng/ml (95%CI: 0.02 ng/ml to 0.28 ng/ml) from baseline to day 2 in the control group and decreased by 0.15 ng/ml (95%CI: 0.01 ng/ml to 0.30 ng/ml) in the thiamine group. The mean differences and 95% confidence intervals from baseline to Time 1 and 2 for both the unadjusted and adjusted models are shown in Table 4.

There was no statistically significant difference between the two groups on change in free fatty acid levels from baseline to day 2 in both the unadjusted (*p* = 0.086) and adjusted (*p* = 0.087) models. Free fatty acid (unadjusted) levels increased by 0.16 mEq/ml (95%CI: 0.05 mE/ml to 0.27 mEq/ml) from baseline to day 2 in the control group and increased by 0.24 mEq/ml (95%CI: 0.13 mE/ml to 0.35 mEq/ml). The mean differences and 95% confidence intervals from baseline to Time 1 and 2 for both the unadjusted and adjusted models are shown in Table 4.

There was no statistically significant difference between the two groups on change in glucose levels from baseline to day 2 in both the unadjusted (*p* = 0.491) and adjusted (*p* = 0.515) models. Glucose (unadjusted) levels decreased (improved) by 48 mg/dl (95%CI: 24 mg/dl to 72 mg/dl) from baseline to day 2 in the control group and decreased by 38 mg/dl (95%CI: 13 mg/dl to 64 mg/dl) in the thiamine group. The mean differences and 95% confidence intervals from baseline to Time 1 and 2 for both the unadjusted and adjusted models are shown in Table 4.

### Length of stay, 30-day Rehospitalization, and mortality

The difference between the control and treatment groups on hospital length of stay was 0 days (95%CI: -1 to 1) for the unadjusted model yielding a *p*-value of 0.71 (Table 5). The difference between the control and treatment groups on percent of patients rehospitalization within 30 days was 6.1% (95%CI: -9.7 to 22.0) for the unadjusted model yielding a p-value of 0.45. All of the covariates fell out of the adjusted models. The difference between the control and treatment groups on percent mortality was 1.6% (95%CI: 0.02 to 6.36) yielding a p-value of 1.00. The small number of deaths (*n* = 3) prevented any further analysis of this outcome measure.

**Table 5:** Morbidity and Mortality outcomes by thiamine supplementation

Measure	Control	Treatment	*p*-value (model)
Length of Stay (mean days)			
Unadjusted (*n* = 117)*	5 (4–6)	5 (4–6)	0.71
30-Day Rehospitalization (%)			
Unadjusted (*n* = 107)*	20.1 (9.2–31.0)	26.2 (15.1–37.4)	0.45
^**^Mortality (%)			
*n* = (111)	1.9 (−1.8 to 5.5)	3.5 (−1.4 to 8.4)	1.00

Estimates include 95%Confidence Intervals.. *P*-values are from the treatment group indicator in the model

^*^Includes design variables: site, diabetes medication (self-report) and NT-proBNP quartile. Deceased patients excluded. (*n* = 3)

^**^Due to the small number of events (Control *n* = 1, Treatment *n* = 2) modeling was not conducted. *P*-value from Fisher exact test

### Post-hoc analyses

All subjects enrolled were adjudicated to have acute heart failure as the primary cause of their dyspnea. The two adjudication reviewers had 100% agreement. We performed a post-hoc assessment of the impact of subject loss at day 2. The above analyses were repeated with day 2 data dropped (see Additional file 1: Table S2b, Additional file 2: Table S3b and Additional file 3: Table S4b). There was minimal impact on the effect sizes observed at time one.

## Discussion

Change in cellular metabolism is thought to play an important role in the development and progression of heart failure. In the healthy adult heart, fatty acids are the primary metabolic substrate; however, in heart failure there appears to be a shift to a greater reliance on glucose as a metabolic substrate. However, there is controversy as to when this shift occurs and whether this shift is a beneficial or harmful adaptation [13–16].

While there are many metabolic changes associated with heart failure, one commonly found alteration is a decrease in pyruvate dehydrogenase function secondary to an in crease in pyruvate dehydrogenase kinase. In animal models, blocking the effect of pyruvate dehydrogenase kinase improves ATP production and cardiac function [17].

Studies with agents that shift energy metabolism from fatty acids to glucose metabolism have been shown to improve ATP production and cardiac function [18, 19]. Although, the effect is not consistent [20, 21].

Patients with heart failure are at risk for thiamine insufficiency [7]. Increased urinary excretion secondary to diuretic use has been shown to deplete serum thiamine [22]. Many patients with heart failure do not meet the recommended dietary allowances and dietary reference intake for many nutrients including thiamine [23]. Thiamine is a necessary cofactor for pyruvate dehydrogenase. It is unknown what the optimum thiamine levels are for pyruvate dehydrogenase activity.

There have been five prior studies evaluating the impact of thiamine treatment for heart failure [24–28]. Seligmann (1991) gave six thiamine deficient patients hospitalized with acute decompensated heart failure intravenous thiamine (100 mg twice a day) for 1 week [24]. On average their NYHA functional class improved by 1 class. LVEF improved in four subjects, remained unchanged in one subject and was not measured in one subject. There was no control group. Pfitzenmeyer (1994) randomized 35 admitted heart failure patients to either thiamine (200 mg /day) or no thiamine for 1 week [25]. They detected no clinical difference between the two groups; however, no details for how this was determined was provided. Shimon [26] randomized 30 patients with stable chronic heart failure to intravenous thiamine (100 mg twice a day) or placebo for 1 week. The authors claimed to show a significant change in LVEF in the thiamine group but not in the placebo group. However, when we re-analyzed their data, there was no significant difference between the two groups, *p* = 0.24. Smithline (2007) randomized 50 patients to a single dose of thiamine (100 mg) or placebo within 30 min of arrival to the emergency department with acute heart failure [27]. There was no difference in admission rate or change in dyspnea after 4 h of treatment. Schoenenberger (2011) performed a randomized two-period -over study in ten patients with chronic stable heart failure [28]. Patients received thiamine (300 mg/day) or placebo for 1 month with a 6-week washout period. They found a significant improvement in LVEF between the two groups with an absolute difference of 3.9%.

On the primary outcome measure, dyspnea as measured in the first position, the thiamine group improved less than the control group. In contrast, dyspnea measured in the second position and dyspnea measured by the PDA did not show any differences between groups. Finally, an objective measurement of peak flow was not different between groups. Thus, with two measures of dyspnea indicating no difference and other objective measures in the study indicating no physiologic impact of thiamine, we suspect that the change in dyspnea as measured in the first position may be artifact and/or not clinically relevant. There is no report of thiamine creating a sensation of dyspnea in the literature. Although, hypothetically, thiamine could increase oxygen consumption, stimulating the respiratory system and thus create a sensation of dyspnea. By a similar mechanism caffeine has been shown to cause dyspnea. Apart from this isolated finding in one of three dyspnea scores, there were no differences between groups on any other parameters.

Our study has several limitations. While we anticipated that a number of patients would have low thiamine levels, this turned out not to be true as only one subject had thiamine deficiency. It is possible that other populations, with lower thiamine levels may yet benefit from thiamine treatment. Supporting this is a study of thiamine supplementation for septic shock that found that patients with low thiamine levels improved with thiamine therapy [29]. We used a dose of thiamine (100 mg) that has been traditionally used to treat thiamine deficiency syndromes such as beriberi and Wernicke-Korsakoff syndrome. Recently, higher dosages have been recommended. In the above mentioned trial in septic shock a 200 mg regimen was used [29]. The optimal dosing regimen for treating thiamine deficient disorders has never been formally studied and the possibility remains that higher dosages may have been necessary to see an effect [30]. Additionally, according to the protocol, thiamine treatment did not begin until the evening of the first in-hospital day. Earlier thiamine treatment may have resulted in different findings. The sample size of our study was relatively small which limits our ability to be definitive about our results. Finally, although alternative measurement techniques of dyspnea have received recent attention, extensive use of and validation these scales has yet to occur [11].

## Conclusions

For patients with mild-moderate acute heart failure without thiamine deficiency, the administration of thiamine did not improve dyspnea. In the primary outcome measure for dyspnea, the control arm had better improvement in dyspnea while two alternative measures of dyspnea and an objective measure of expiratory airflow showed no difference between groups. This pilot study does not support the use of thiamine for the treatment of subjects with acute heart failure syndrome without thiamine deficiency.

## Additional files

Additional file 1: **Table S2b.** Whole Blood Thiamine divided by Hemoglobin (nmol/L/g Hgb) by Time (*n* = 116). Analysis excludes Time 2. (DOCX 15 kb)

Additional file 2: **Table S3b.** Physiologic outcomes by thiamine supplementation over time. Analysis excludes Time 2. (DOCX 20 kb)

Additional file 3: **Table S4b.** Mean difference and 95% CI over time compared to baseline. Analysis excludes Time 2. (DOCX 19 kb)

## Abbreviations

ATP: Adenosine triphosphate
FFA: Free fatty acids
LVEF: Left ventricular ejection fraction
NT-proBNP: N-terminal prohormone of brain natriuretic peptide
PDA: Provocative Dyspnoea Assessment
PEFR: Peak expiratory flow rate
VAS: Visual analog scale
VAS-PDA: Combined scaled VAS scores from the three body positions
