# Supplementary material for: Supplemental thiamine for the treatment of acute heart failure syndrome: a randomized controlled trial
Source: BMC Complement Altern Med. 2019 May 6;19:96. doi: 10.1186/s12906-019-2506-8 (PMC6501378; doi:10.1186/s12906-019-2506-8)
Supplement: Supplementary file 1 — Table S2b. Whole Blood Thiamine divided by Hemoglobin (nmol/L/g Hgb) by Time (n = 116). Analysis excludes Time 2. (DOCX 15 kb) [file 12906_2019_2506_MOESM1_ESM.docx]

SUPPLEMENTAL ANALYSIS

(These analyses exclude Time 2)

Table S2b

Whole Blood Thiamine divided by Hemoglobin (nmol/L/g Hgb) by Time (n=116)

|  | Control | Treatment | Difference | *p-value*  *(Sidak adjusted)* |
| --- | --- | --- | --- | --- |
| Baseline | 10.72 (5.26 – 16.18) | 10.75 (5.66 – 15.85) | 0.03 (-8.49 – 8.55) | 1.0 |
| Time 1 | 10.36 (4.96 – 15.77) | 51.67 (46.53 – 56.80) | 41.30 (32.79 – 49.81) | <0.001 |
