# Supplementary material for: Supplemental thiamine for the treatment of acute heart failure syndrome: a randomized controlled trial
Source: BMC Complement Altern Med. 2019 May 6;19:96. doi: 10.1186/s12906-019-2506-8 (PMC6501378; doi:10.1186/s12906-019-2506-8)
Supplement: Supplementary file 2 — Table S3b. Physiologic outcomes by thiamine supplementation over time. Analysis excludes Time 2. (DOCX 20 kb) [file 12906_2019_2506_MOESM2_ESM.docx]

Table S3b

Physiologic outcomes by thiamine supplementation over time

| Measure | Baseline | | Day 1 | | *p-value (model)* |
| --- | --- | --- | --- | --- | --- |
|  | Control | Treatment | Control | Treatment |  |
| VAS-1^st^ Position (mm) |  |  |  |  |  |
| Unadjusted (n=116)* | 31 (24-38) | 26 (19-33) | 17 (12-22) | 22 (16-28) | *0.004* |
| Adjusted (n=104)**^a,b^ | 29 (28-31) | 28 (26-29) | 15 (10-20) | 24 (19-28) | *0.006* |
| VAS-2^nd^ Position (mm) |  |  |  |  |  |
| Unadjusted (n=99)* | 31 (23-38) | 25 (17-32) | 18 (12-24) | 19 (14-24) | *0.093* |
| Adjusted (n=83)** ^b,c^ | 29 (27-31) | 27 (25-28) | 16 (11-21) | 21 (17-25) | *0.086* |
| VAS-PDA (mm) |  |  |  |  |  |
| Unadjusted (n=116)* | 103 (78-128) | 80 (59-101) | 78 (54-102) | 58 (39-77) | *0.806* |
| Adjusted (n=97)** ^a,c^ | 82 (75-89) | 76 (70-82) | 61 (39-83) | 58 (45-72) | *0.807* |
| PEFR (L/min) |  |  |  |  |  |
| Unadjusted (n=113)* | 203 (180-226) | 174 (152-196) | 206 (183-229) | 163 (142-185) | *0.099* |
| Adjusted (n=103)** ^b,d^ | 189 (181-197) | 186 (178-193) | 195 (186-203) | 174 (167-182) | *0.033* |
| NT-proBNP (ng/ml) |  |  |  |  |  |
| Unadjusted (n=113)* | 0.42 (0.32-0.52) | 0.51 (0.35-0.68) | 0.45 (0.30-0.61) | 0.41 (0.28-0.53) | *0.128* |
| Adjusted (n=100)** ^c^ | 0.47 (0.44-0.50) | 0.48 (0.44-0.51) | 0.44 (0.36-0.52) | 0.34 (0.26-0.42) | *0.126* |
| FFA (mEq/ml) |  |  |  |  |  |
| Unadjusted (n=113)* | 0.46 (0.38-0.54) | 0.47 (0.39-0.56) | 0.68 (0.62-0.74) | 0.65 (0.59-0.72) | *0.567* |
| Adjusted (n=102)** ^a,b^ | 0.46 (0.42-0.49) | 0.46 (0.42-0.49) | 0.69 (0.62-0.76) | 0.63 (0.56-0.70) | *0.390* |
| Glucose (mg/dl) |  |  |  |  |  |
| Unadjusted (n=117)* | 166 (146-186) | 155 (135-174) | 121 (110-132) | 111 (101-122) | *0.883* |
| Adjusted (n=113)** | 161 (151-170) | 158 (148-167) | 119 (107-131) | 113 (98-128) | *0.866* |
| Values are means with 95%CI. P-values are from a test of the treatment*time interaction term in the mixed model.  *Includes design variables: site, diabetes medication (self-report) and NT-proBNP quartile  **Additional adjustments for: baseline values of the outcome, thiamine, BMI^a^, LVEF>=50% ^b^, PEFR ^c^, systolic BP ^d^ | | | | | |
