# Supplementary material for: Supplemental thiamine for the treatment of acute heart failure syndrome: a randomized controlled trial
Source: BMC Complement Altern Med. 2019 May 6;19:96. doi: 10.1186/s12906-019-2506-8 (PMC6501378; doi:10.1186/s12906-019-2506-8)
Supplement: Supplementary file 3 — Table S4b. Mean difference and 95% CI over time compared to baseline. Analysis excludes Time 2. (DOCX 19 kb) [file 12906_2019_2506_MOESM3_ESM.docx]

Table S4b

Mean difference and 95% CI over time compared to baseline

| Measure | Delta Baseline to Time 1 | |
| --- | --- | --- |
|  | Control | Treatment |
| VAS-1^st^ Position (mm) |  |  |
| Unadjusted* | -14 (-20 to -8) | -4 (-9 to 1) |
| Adjusted**^a,b^ | -15 (-21 to -8) | -4 (-10 to 2) |
| VAS-2^nd^ Position (mm) |  |  |
| Unadjusted* | -12 (-20 to -5) | -6 (-11 to -1) |
| Adjusted** ^b,c^ | -13 (-21 to -5) | -6 (-11 to -1) |
| VAS-PDA (mm) |  |  |
| Unadjusted* | -25 (-53 to 2) | -22 (-39 to -4) |
| Adjusted** ^a,c^ | -21 (-51 to 9) | -18 (-34 to -1) |
| PEFR (L/min) |  |  |
| Unadjusted* | 3 (-11 to 16) | -11 (-23 to 2) |
| Adjusted** ^b,d^ | 5 (-7 to 18) | -11 (-23 to 1) |
| NT-proBNP (ng/ml) |  |  |
| Unadjusted* | 0.03 (-0.14 to 0.21) | -0.11 (-0.22 to 0.00) |
| Adjusted** ^c^ | -0.03 (-0.13 to 0.06) | -0.14 (-0.26 to -0.02) |
| FFA (mEq/ml) |  |  |
| Unadjusted* | 0.22 (0.12 to 0.31) | 0.18 (0.08 to 0.29) |
| Adjusted** ^a,b^ | 0.23 (0.13 to 0.33) | 0.17 (0.06 to 0.28) |
| Glucose (mg/dl) |  |  |
| Unadjusted* | -45 (-67 to -23) | -43 (-70 to -17) |
| Adjusted** | -42 (-63 to -20) | -44 (-71 to -17) |
| Values are mean difference and Sidak adjusted 95% Confidence Intervals  *Includes design variables: site, diabetes medication (self-report) and NT-proBNP quartile  **Additional adjustments for: baseline values of the outcome, thiamine, BMI^a^, LVEF>=50% ^b^, PEFR ^c^, systolic BP ^d^ | | |
